# Die intraoperative Strahlentherapie (IORT) als mögliche Alternative zum vollständigen Verzicht auf eine adjuvante Radiotherapie nach brusterhaltender Operation bei älteren Patientinnen mit niedriger Risikokonstellation

**DOI:** 10.1007/s00066-024-02203-z

**Published:** 2024-02-07

**Authors:** Mathias Alexander Sonnhoff, Robert Maximilian Blach, Robert Michael Hermann

**Affiliations:** 1 Medizinische Hochschule Hannover, Klinik für Strahlentherapie und Spezielle Onkologie, Hannover, Deutschland Carl- Neuberg-Straße 1, 30625; 2 Arbeitsgruppe junge DEGRO der Deutschen Gesellschaft für Radioonkologie e. V. (DEGRO), Berlin, Deutschland; 3 Zentrum für Strahlentherapie und Radioonkologie, Gröpelinger Heerstr. 406–408, 28239 Bremen, Deutschland

## Hintergrund und Fragestellung

Im Rahmen des Brusterhalts bei lokal begrenztem Mammakarzinom spielt die adjuvante Radiotherapie (RT) eine wichtige Rolle. Inzwischen sind diverse Fraktionierungsoptionen als auch verschiedene Zielvolumenkonzepte und Therapietechniken einzeln oder in Kombination etabliert worden. Der Stellenwert der intraoperativen RT (IORT) in diesem Zusammenhang ist in zwei großen Studien geprüft worden [1, 2]. Doch obwohl die RT eine lokal hocheffektive Therapie mit einem sehr günstigen Nebenwirkungsprofil ist, gerät sie durch Studien wie PRIME II unter Druck, in der eine niedrige Lokalrezidivrate bei günstiger Risikokonstellation trotz Verzichts auf die RT beschrieben wird [3]. Dennoch sollten, bevor die Diskussion über Deeskalation weiter vorangetrieben wird, die weiteren Optionen der lokalen RT ausgelotet werden. Die hier vorgestellte Publikation aus Brasilien liefert in diesem Kontext einen weiteren Beitrag zur Diskussion der Indikationsbreite der IORT und der lokalen Adjuvanz.

## Patienten und Methodik

In einer prospektiven Phase-2-Studie eines brasilianischen Zentrums wurden von 2004 bis 2016 *n* = 209 Patientinnen rekrutiert. Eingeschlossen wurden Patientinnen über 40 Jahre mit einem infiltrierendem duktalen Karzinom (NOS) kleiner als 3 cm, Ausschluss einer Multifokalität im Brust-MRT und negativem Sentinel-Lymphknoten im Schnellschnitt. Nach R0-Resektion wurde intraoperativ eine Schutzplatte zwischen dem Zielparenchym und der Thoraxwand platziert, zur Definition des dorsalen Rands des Zielvolumens. Unter Allgemeinanästhesie und Wahrung der infektiologischen und strahlenschutzrelevanten Kautelen wurden 21 Gy Elektronenbestrahlung mit einem „nondedicated linear accelerator“ (ndLINAC) appliziert [4].

## Ergebnisse

Über 50% der Patientinnen waren älter als 60 Jahre, nur ca. 16% hatten eine Tumorausdehnung > 2 cm, in nur 2% zeigte sich in der histologischen Aufarbeitung der Sentinel-LK eine makroskopische Metastasierung. 30% hatten ein Grading III°, 10% hatten eine lymphovaskuläre Invasion. 63% waren luminal A, 19% luminal B und 12% luminal/„HER2 enriched“.

Das mediane Follow-up umfasste 145 Monate (Range 12,8–187,1 Monate). Nach einem Nachbeobachtungszeitraum von 10 Jahren waren 14 Patientinnen (6 tumorbedingt) verstorben. Es traten 15 lokale und 3 regionale Rezidive auf (75% der Rezidive erst nach > 10 Jahren).

Insgesamt betrug die 15-Jahres-Rezidivrate 7,2 %. In der Untergruppe der Luminal-A-Tumoren lag die Rezidivrate bei 4,6 %, in der Untergruppe der Luminal-B-Tumoren hingegen bei 12,5 %.

## Schlussfolgerung der Autoren

Diese Form der IORT ist bei ausgewählten Patientinnen anwendbar.

## Kommentar

TARGIT [1] und ELIOT [2] sind die beiden großen Leitstudien zur IORT als adjuvante Therapie im brusterhaltenden Therapieansatz des lokal begrenzten Mammakarzinoms.

Während der IORT-Arm der TARGIT-Studie [1] keinen Unterschied zwischen der „whole breast irradiation“ (WBRT) und der IORT zeigte, war in der ELIOT-Studie [2] die IORT der konventionellen WBRT unterlegen. In der Subgruppenanalyse der ELIOT-Studie lag die 5‑Jahres-Rezidivrate bei Luminal-A-Histologie nach IORT bei 1,5 % (im gesamten Kollektiv bei 4,4 %; [2]). Im Rahmen der hier dargestellten Untersuchung traten im Luminal-A-Kollektiv in 4,6 % Rezidive auf [5].

Obwohl die hier vorliegende Untersuchung als einarmige Untersuchung ohne Kontrollgruppe angelegt war, bietet sich immerhin eine Nachbeobachtung von bis zu 15 Jahren. Somit lässt sich die Langzeitcharakteristik der Rezidive beschreiben. Interessanterweise traten 75% der Rezidive erst 10 Jahre nach der lokalen Therapie auf. Auch in der PRIME-II-Studie wurde nach 10 Jahren [3] bei Verzicht auf die lokale adjuvante Therapie nach brusterhaltender Operation kein Plateau erreicht. Dies lässt den Schluss zu, dass nach einer unzureichenden oder gänzlichen Verzicht auf eine lokale Adjuvanz ein signifikantes Rezidivrisiko über das 10-Jahres-Intervall hinaus bestehen bleibt. Eine retrospektive Auswertung von 3171 Patientinnen aus dem Münchener Tumorregister konnte – ähnlich wie PRIME II – darstellen, dass eine adjuvante antihormonelle Therapie eine mögliche Alternative zur Sicherung der lokalen Kontrolle bietet. Allerdings zeigte in dieser Analyse die alleinige RT nach brusterhaltender Operation (ohne antihormonelle Therapie) noch bessere Lokalkontrollraten als die alleinige antihormonelle Therapie [6].

Als Erklärung für diese Beobachtung kann angeführt werden, dass die Wirksamkeit einer Therapie ihre Einnahme voraussetzt. Die Einnahme-Compliance der antihormonellen Therapie ist besonders bei älteren Patientinnen nicht immer gegeben [7]. Unter solchen Umständen stellt die RT eine Alternative zur alleinigen antihormonellen Therapie bei älteren Patienten mit niedrigem Risikoprofil dar. Der Einsatz der IORT in diesem Setting als strahlentherapeutische Behandlungsoption kann dabei in Hinblick auf Aufwand (Verlängerung der OP-Zeit, Kosten etc.) und Komplexität bei der Etablierung kritisch hinterfragt werden. Als zwischenzeitlich flächendeckende, wenig aufwendige und gut verträgliche strahlentherapeutische Alternativen stehen mittlerweile die hypofraktionierte Teilbrustbestrahlung bis hin zu Kurzzeitkonzepten nach „FAST“ für Patientinnen, die nur einen minimalen Zeitaufwand wünschen, zur Verfügung [8, 9].

### Fazit

Eine Therapiedeeskalation für ältere Patientinnen mit niedrigem Risiko muss nicht im gänzlichen Verzicht auf die Radiotherapie resultieren. Die IORT kann hier eine Alternative sein.

*Mathias Alexander Sonnhoff, Robert Maximilian Blach, Robert Michael Hermann*
